# Therapeutic Outcomes of Photobiomodulation in Cancer Treatment-induced Oral Mucositis: A Systematic Review

**DOI:** 10.4317/jced.60710

**Published:** 2023-09-01

**Authors:** Rebeca Sánchez-Martos, Wissal Lamdaoui, Santiago Arias-Herrera

**Affiliations:** 1 Universidad Europea de Valencia. Faculty of Health Sciences. Department of Dentistry

## Abstract

**Background:**

This systematic review was performed to analyze the therapeutic efficacy of photobiomodulation (PBM) in managing oral mucositis (OM) that appears in this context.

**Material and Methods:**

The search strategy of the systematic review was conducted according to PRISMA guidelines. The eligibility criteria according to PICO process has been defined as follows: Population (P): adult patients with head and neck cancer; Intervention (I): PBM; Comparison (C): placebo group; Outcome (O): pain, oral quality of life (QoL), evolution of the grade OM and pain. The set criteria for inclusion were peer-reviewed articles.

**Results:**

The following database were searched from November 2021 to February 2022, for clinical trials: Pubmed, Scopus and Cochrane. From 296 records, 10 studies were included involving in the systematic review. Data from 759 patients who received chemoradiotherapy were analyzed. These studies used different classifications for oral mucositis (WHO, NCI, RTOF/EORTC), pain (VAS) and quality of life (EORTC QLQ-C30, UW-QOL (v4), FACT-HN). PBM therapy protocol used five different lasers (GaAlAs, InGaAlP, He-Ne, diode laser, red and near-IR LED probe) with wavelengths ranging from 632,8nm to 850nm. Pain evaluation in was based on the visual analogue scale (VAS) mainly. Prophylactic PBM was effective as it reduced the incidence of grades 3-4 and reduced the overall mean grade of OM during the chemoradiotherapy course compared to the control group. On the other hand, when PBM was used for treatment purposes, it decreased the mean duration of OM compared to the placebo arm.

**Conclusions:**

PBM reduced the incidence of more severe grade of OM induced by chemoradiotherapy. Also, PBM therapy reduced the mean duration of severe OM, mean pain scores and subsequently improved QoL.

** Key words:**Oral mucositis, photobiomodulation, low-level laser therapy, chemotherapy, radiotherapy.

## Introduction

**Figure 1:**
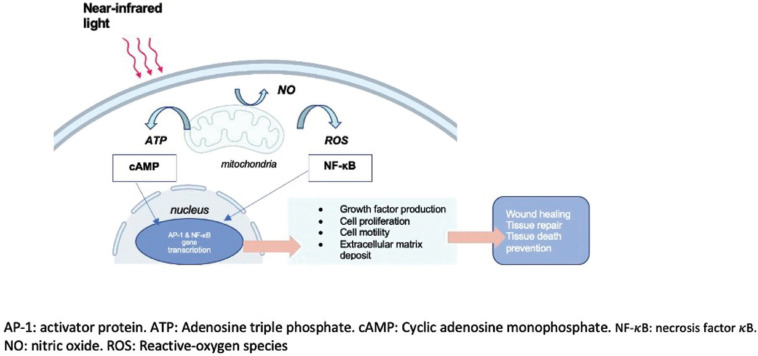
Mechanism of phtobiomodulation.

Photobiomodulation (PBM), formerly known as “low-level laser therapy” (LLLT) or also cold therapy was brought in for the first time in medicine by Endre Mester in 1967 reporting its stimulatory (wound healing) and inhibitory (pain treatment) effects in biological tissues (1). The PBM is based on the process illustrated in (Fig. 1) whereby the light is absorbed and produces effects on the biological systems. Several lines of evidence suggest that PBM acts on mitochondrial cytochrome C oxidase (CCO), which in turn enhances secondary cell-signaling pathways and results in increasing the levels of ATP, cAMP, and reactive oxygen species (ROS) (1,2). Nowadays, PBM, refers to low-power laser therapies since PBM comprises broadband lights, LEDs, and lasers, which all of them establish a wide range of electromagnetic radiations (3). PBM has played an influential role oral medicine field in both *in vitro* and *in vivo* studies.

**Figure 2:**
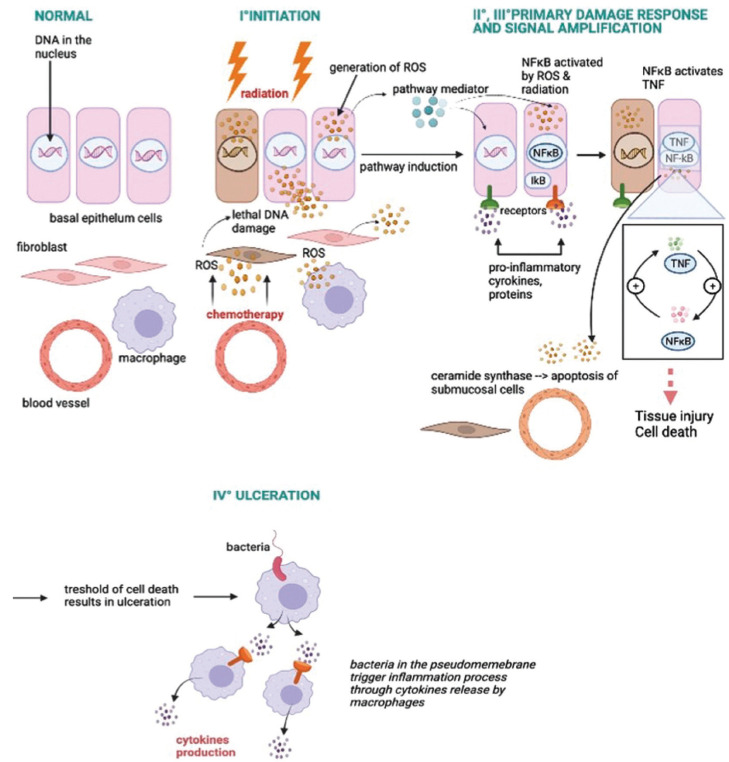
Physiopathology of oral mucositis.

Oral mucositis (OM) caused by radiation of chemoradiotherapy is one key example of oral pathology where PBM has been studied as a therapeutic tool (6). Oral mucositis (OM) or also called in the literature “mucosal barrier injury” is characterized by widespread erythema, ulceration, and soreness. It is a condition that involves pain and oral discomfort, ranging from mild to severe, and hence affects severely patients’ nutritional intake and overall comprises quality of life (Fig. 2) illustrates the pathophysiology of oral mucositis, based on damage to the cellular DNA of the affected area. OM is an ailment that remains the most frequent complication in head-neck cancer patients. It arises from cytotoxic effects of therapies for malignant lesions such as radiotherapy or chemotherapy (7).

The prevalence of OM in head and neck cancer patients treated with chemotherapy and radiotherapy can reach up 100% of cases. OM provoked by theses therapies can cause unbearable mouth discomfort to the patients accompanied with pain, swelling, difficult oral hygiene, and reduced oral intakes. The mucosa barrier is debilitated, thus leading to local or systemic infections and poor quality of life of these patients.

It exists three most frequently used scales among a variety for grading OM. The oral toxicity scales most commonly applied are the following: Radiotherapy Oncology Group (RTOG), World Health Organization (WHO), and National Cancer Institute Common Terminology Criteria for Adverse Events (CTCAE). Table 1 illustrates the scale of each classification, dividing the severity of OM into four grades.

**Table 1:**
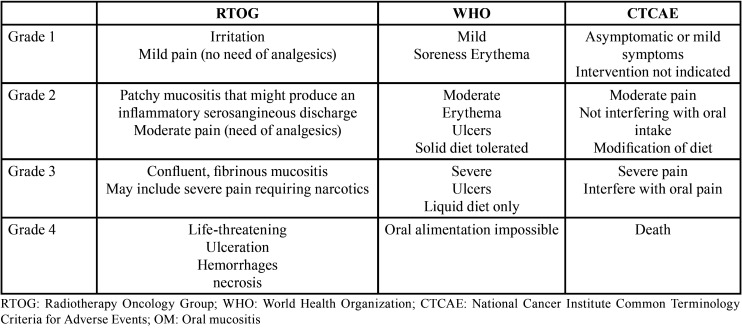
RTOG, WHO, and CTCAE toxicity scales of OM.

In general, the management of OM lesions is based on the symptomatology of the patient as there is no gold standard treatment of it. Current systematic review reports the power of use and benefits achieved of PBM regarding OM. As a result, PBM is found to be a drug-free and less invasive option that is better accepted by the patients when one considers the results obtained.

The general objective of the present systematic review is to analyze the therapeutic

efficacy of PBM in managing OM that appears in head and neck patient treated with radiotherapy and chemotherapy.

## Material and Methods

-Inclusion and exclusion criteria

The eligibility criteria according to PICO process has been defined as follows: Population (P): adult patients with head and neck cancer; Intervention (I): photobiomodulation; Comparison (C): placebo group; Outcome (O): pain, oral quality of life (QoL), evolution of the grade OM.

Subsequently, our research question would be as follows: in head and neck cancer patients undergoing radiotherapy or chemotherapy, does photobiomodulation helps improving the grade of OM as well as pain and quality of life compared to placebo group? 

The present systematic review included studies conducted on humans published in English from November 2011 to November 2021. We also included studies if they met the standards of clinical trials and random clinical trial (RCT), with placebo or control group, usual care, patients with OM, patients who undergone radiotherapy or chemotherapy for the treatment of head and neck cancer, together with photobiomodulation for either treatment or prevention of OM.

Exclusion criteria were: animals, children, patients who received stem-cell transplantation, patients with other oral consequences due to radiotherapy or chemotherapy, non-English articles.

-Search strategy

The planning and preparation of the study has followed the protocols established in the PRISMA guidelines for the preparation of systematic reviews.

A search of articles was carried out in the following databases: Scopus, Pubmed, and Cochrane library; between November 2021 and January 2022. The search strategy included the keywords “low-level laser therapy”,” LLLT”, “oral mucositis”. These terms combined with the Boolean operator “AND/OR” to obtain the articles.

-Selection process of the studies

Titles and abstracts from the three databases were downloaded to Zotero software. Zotero was used to import the reference data and to remove duplicate records. Two reviewers (LW, GFF) screened independently titles and abstracts. Disagreements regarding inclusion were resolved by mutual consensus of both reviewers. Studies that satisfied the eligibility criteria were included through full-text assessment.

-Data extraction and analysis

The following data were obtained from each eligible study: author, year of publication, sample size, type of laser, wavelength, assessment (OM, pain and QoL), OM grades 0-1 and 2-4 in control group (CT) and intervention group (IG), pain grading (VAS) in CG and IG, QoL in CG and IG.

-Quality of evidence evaluation and risk of bias assessment

To assess the overall quality of the evidence, we used the CASPe (Critical Appraisal Skills Program Español) checklist. The CASPe guide for clinical trials is made of eleven questions, three first ones of which are designed to help us to eliminate the less relevant articles. As a matter of fact, if the first three answers are “yes”, then it is worth continuing with the next questions.

-Case definitions

Diode laser therapies: There is no consensus on a gold standard protocol for laser treatment for oral mucositis. We will be discussing two main concepts in this systematic review.

Laser therapy (LT): This therapy is based on the conversion of light energy into thermal energy, increasing the temperature in the tissues and producing injuries that will depend on the degrees reached. Depending on the power at which the laser is used in this therapy, bactericidal, cutting and coagulation effects as well as cellular biostimulation will be obtained (3-5).

Oral mucositis (OM): is characterized by widespread erythema, ulceration, and soreness. It is a condition that involves pain and oral discomfort, ranging from mild to severe, and hence affects severely patients’ nutritional intake and overall comprises quality of life. OM is the most frequent complication in head-neck cancer patients arising from cytotoxic effects of CRT (7).

## Results

-Study selection

Initially, the data base searches identified a total of 274 articles (Fig. 2): PubMed 40 articles, Scopus 124 articles and Cochrane 115 articles., manual search 1 article. After removing all duplicates with Zotero, a total of 169 articles were retrieved for title and abstract evaluation. At the end of the previous process of selection, 34 articles were identified and evaluated independently for analysis of their full text by two reviewers. A total of 10 relevant articles were selected for the purpose of this systematic review. Overall, a total number of 759 patients were randomly assigned in the included studies.

-Description of study characteristics

A total of 10 randomized clinical trials included for the purpose of this systematic review. Overall, a total number of 759 patients were randomly assigned in the included studies. The duration time of PBM was ranging from 10 seconds (9-11) to 125-145 seconds (12-14). There are mainly five types of lasers: GaAlAs (9,10), InGaAlP (11,12), He-Ne (12-15), diode laser (16), red and near-IR LED probe (17). The wavelengths used were: 660nm (9,10,12,16), 685 (11,12), 682,5nm (12-14) and 658nm (15) and 34x660 nm (red) and 35x850nm (near-IR) (17). The energy density used was 2.5J/cm2 (9), 2J/cm2 (11), 3.5J/cm2 (12), 3J/cm2 (13,14), 4J/cm2 (11,15). PBM delivery protocol consisted of 5 consecutive day/week from Monday to Friday going from the first to the last session of chemoradiotherapy. We found that two of them looked at both prevention and treatment of OM lesions (24,26). Four of them looked at exclusively preventing the severity of OM (9,11,17). Eventually, four of them aimed to heal OM lesions caused by chemoradiotherapy (10,11,13,15). Tables  Table 2,  Table 2 cont. and  Table 3,  Table 3 cont. summarize the characteristics of the studies included in the review.

**Table 2:**
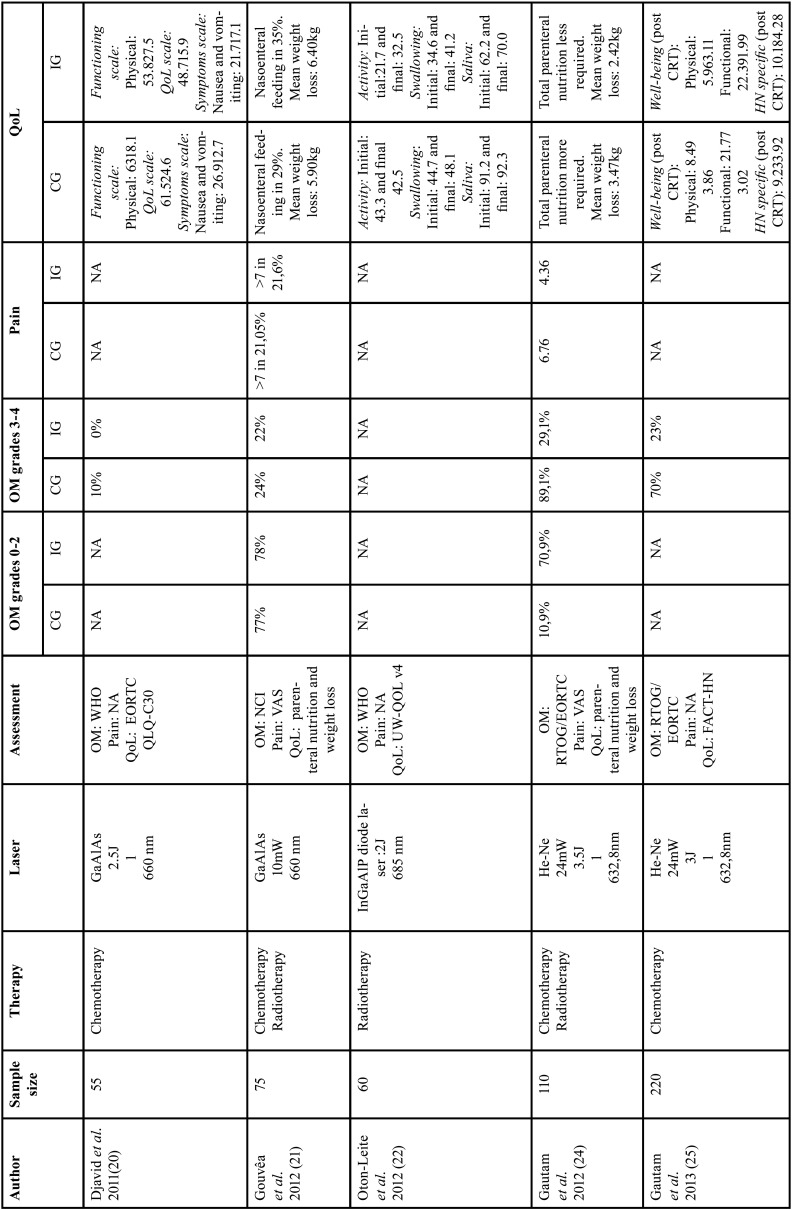
General characteristics and PBM therapy of included studies (part 1).

**Table 2 cont.**
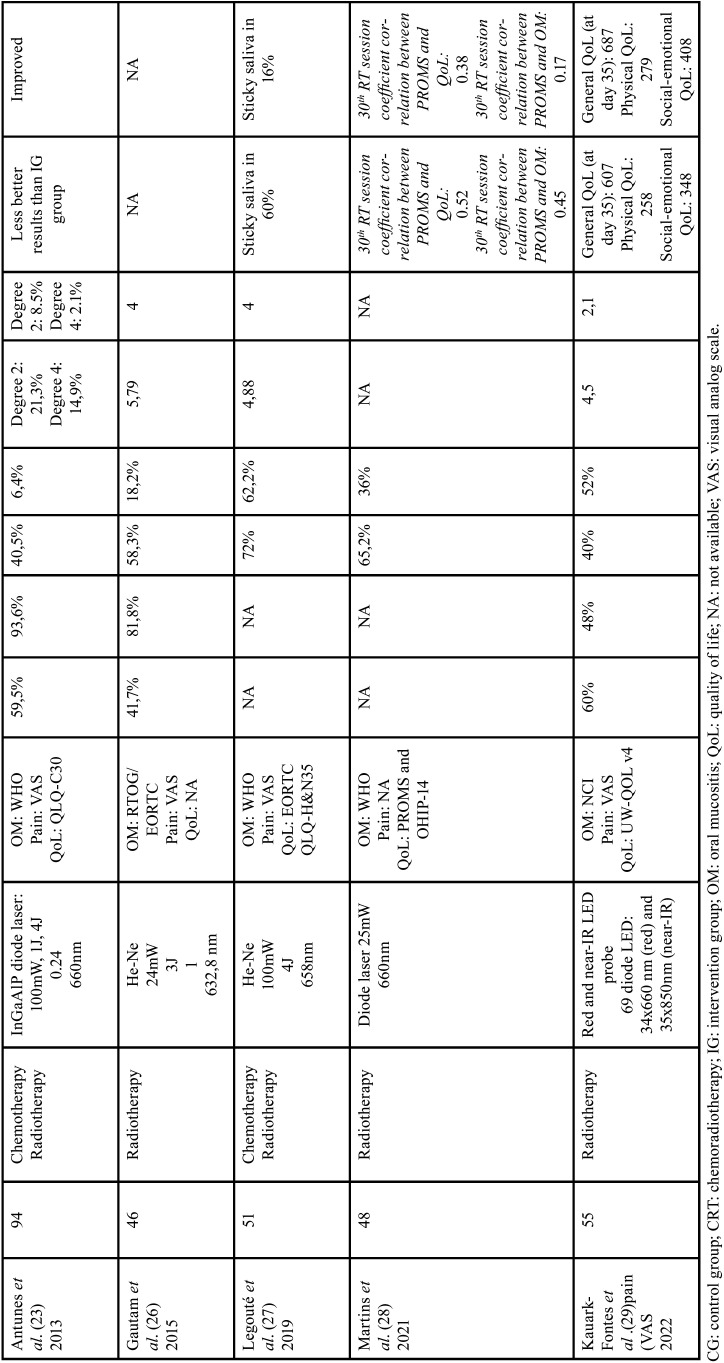
General characteristics and PBM therapy of included studies (part 1).

**Table 3:**
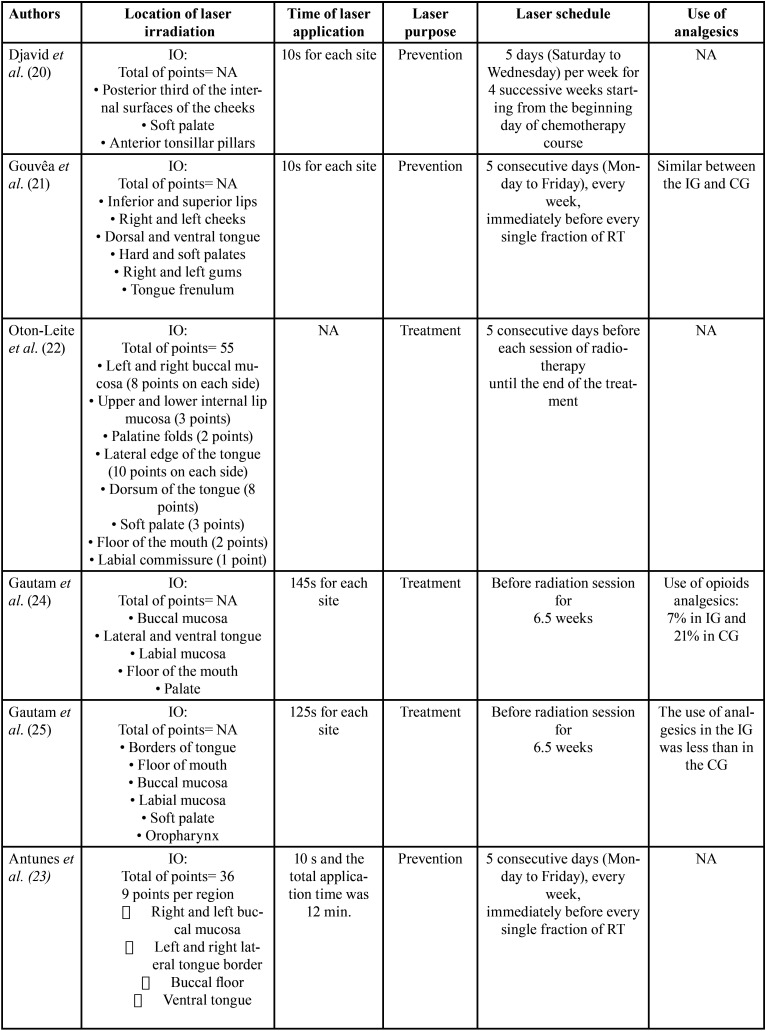
General characteristics of the included studies (part 2).

**Table 3:**
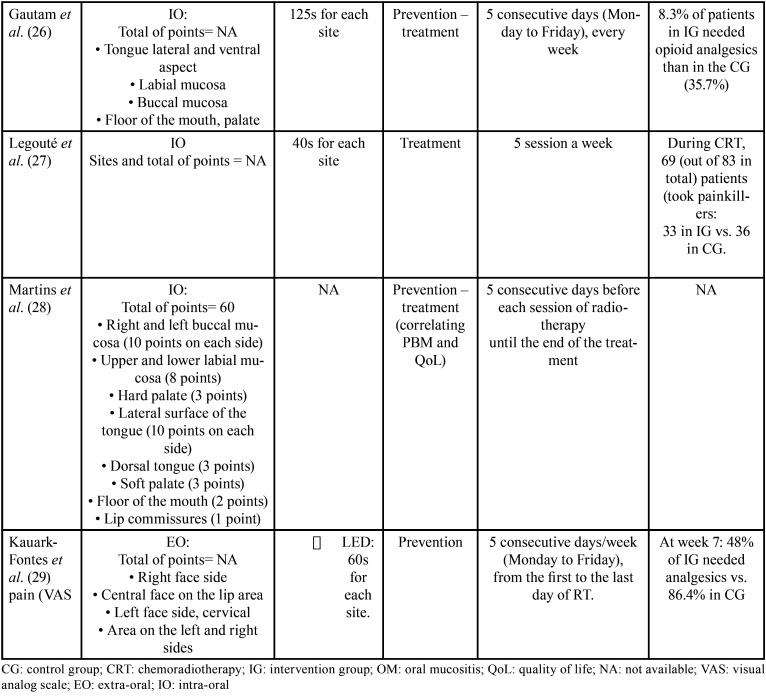
General characteristics of the included studies (part 2).

-Risk of bias and quality of evidence

The risk of bias is summarized in annex 2 using CASPe guide. CASPe questionnaire for randomized clinical trials is made of 12 questions by which we can either answer by “yes”, “no” or “can’t tell. The first questions help us in the process of screening, hence the articles obtaining a greater number of “yes” to these questions are worth proceeding with the following ones. The results showed that the mean quality levels of the 10 articles were considered to be high with overall risk of bias low.

-Synthesis of the results

Laser protocol. This study includes five types of lasers: GaAlAs (8,9), InGaAlP (10,11), He-Ne (12-15), diode laser (16), red and near-IR LED probe (17). The wavelengths used were from 658nm (15) to 35x850nm (near-IR) (17). PBM delivery protocol consisted of 5 consecutive day/week from Monday to Friday going from the first to the last session of chemoradiotherapy.

Pain. Six included studies (2,11,12,14,15,17) used VAS (visual analog scale) instrument for measuring pain. In fact, in the study of Gautam *et al*. (12) the mean VAS score of the PBM group was lower (4.36) than the placebo group (6.76). Kauark *et al*. (17) recorded lower pain score in PBM group of 2.1, which refers to mild pain whereas higher scores of pain were observed in the placebo group (4.5).

Oral mucositis. Four trials (8,10,11,15,16) used WHO grading. Two studies used NCI (9,17) and three articles used RTOG/EORTC criteria (12-14). The literature describes a mean of 19,4% of patients treated with PBM affected by OM severe grades. In the control group (sham laser group), the mean of OM severe grades’ reaches 52,1%. Gautam *et al*. (12) obtained 70,9% of the patients presenting OM with a grade ranging from 0 to 2 and 29,1% with grade 3-4.

QoL. Two articles (8,11) used EORTC QLQ-C30 instrument to assess QoL. Two included studies (10,17) used UW-QOL (v4) questionnaire. One article (13) used FACT-HN questionnaire. In the study conducted by Djavid *et al*. (8) using EORTC QLQ-C30, showed that there were no significant difference between the CG and IG in either the functional or symptoms scales after chemoradiotherapy (CG: functioning scale physical: 63±18.1; IG: functioning scale physical: 53.8±27.5).

## Discussion

-Discussion of the methodology 

In general, the risk of bias of the included studies was classified as low (favorable) according to CASPe checklist (using the newly updated version) in the four sections that do constitute the questionnaire. As our systematic review include mainly randomized clinical trials, it leads to the robustness of the results.

In 2018, the WALT meeting, members of this association agreed that the optimum dose for curing OM was 5J/cm2 and laser energy delivery ranging from 10 and 150mW/cm2 (18). The choice of laser wavelengths applied to reach positive effect in this systematic review, ranged from 632,8nm to 850nm.

-Discussion of the results

-Reduction of pain

Among the included studies, five (11-13,15,17) did address the reduction of pain severity (VAS>7) through the selective inhibition of peripheral pain receptors brought about the application of PBM. Patients were evaluated using the VAS and most studies reported reduction of pain due to the use of PBM. However, in the study of Gouvêa *et al*. (9) there was barely any difference between the two arms (CG and IG) as PBM did not help to control pain. Several reasons might be involved for this heterogeneity, first, in this study patients were administered pain killers, second, patients selected already had OM lesions at advanced stages which involves much more intense and severe pain that probably PBM was not strong enough to decrease it.

Thus, the use of PBM on OM lesions has shown very promising results regarding the management of pain as some authors (12-15,17) reported a decrease of pain scores using the VAS.

-Prevent the evolution of oral mucositis

Heterogeneity was detected in our systematic review because several scoring systems of OM were used that may lead discrepancies between studies. In the evaluation of OM using the WHO scale, Djavid *et al*. (8), Antunes *et al*. (11), Legouté *et al*. (15) and Martins *et al*. (17), PBM had very efficient result at the end of CRT sessions as it delays the exacerbation of OM towards higher grades. According to RTOG/OERTC grading, used in Gautam *et al*. in 2012, 2013 and 2015 (12-14), also slowed the impact on OM severity. The assessment of OM according to the NCI grading of Gouvêa *et al*. (9) and Kauark *et al*. (17) showed that PBM was not effective. In the study of Gouvêa *et al*. (9) and Kauark *et al*. (17), the rate of patients with OM grade 3-4 was very similar to the CG which could be due to the important number of CRT interruptions in the sham-laser group.

As a matter of fact, the use of PBM therapy helps in slowing down the process of severe OM progression compared to the sham group. Hence, the application of LLLT restrains the evolution of OM onto more severe and acute grades over time.

-Quality of life after treating oral mucositis

The properties of PBM offer many benefits in regard to OM and its management. Indeed, PBM has analgesic properties, decreases the inflammation, and helps in reducing the grade severity of OM.

Martins *et al*. (16) was the only study to use the PROMS questionnaire and showed, as a matter of fact, that higher PROMS scores were associated with severe OM in the placebo group leading to reduced QoL. At final stages of radiotherapy in PBM group, the correlation coefficients between PROMS and OM severity were low, which could suggest the efficacy of PBM treatment (16). In the study of Oton-Leite *et al*. (10), the results obtained using UW-QOL (v4) tool inferred that local application of PBM improves QoL and oral functional status. Kauark-Fontes *et al*. (17) also used UW-QOL (v4) questionnaire and reported similar results. In the study of Djavid *et al*. (8), the heterogeneity of the results could be due to the low percentage of patients having OM grade 3-4 (severe) in both IG and CG. The irrelevant results regarding QoL reported in the study of Gouvêa *et al*. (9) could be explained by the comparable rate of patients in IG and CG experiencing OM severe grades, which could be a reason why similar results in both arms were described.

Overall, all the results gathered in this present systematic review are consistent with the litterature. In the systematic review and meta-analysis of Peng *et al*. (19) published in 2020, a total of 30 RCTs were included in order to study therapeutic and prophylactic effect of PBM in patients undergoing chemoradiotherapy. Though this review, the authors found that PBM tremendously reduced the duration of OM compared to the placebo group when it was used for treatment purposes. Moreover, they could conclude that the prophylactic use of PBM prevented the evolution of OM onto more severe grades. Those findings are; as a matter of fact; identical to ours. Another systematic review and meta-analysis of Bjordal *et al*. (18) studied the effect of PBM on OM in the same target population and the conclusions were indeed in favor of PBM as they found out that it reduced both the severity and pain duration of OM.

Although the effects of PBM have been already systematically reported and discussed, this systematic review presents limitations. First, it included studies with different OM scoring systems which made our analysis difficult. Besides, there was a lack of standardization in QoL evaluation and questionnaires. Finally, differences in laser setting protocol among the included study is also considered as part of the limitations of our study. Therefore, all these elements obtained in the studies make the reproducibility of the results difficult. The heterogeneity in the methodology hinders the comparison, thus, our results should be taken with caution.

PBM therapy is effective in preventing the incidence of OM since our findings show that the prophylactic use of PBM decreases the risk of severe OM grades. The use of PBM for treating OM lesions is efficient as our results indicate that PBM therapy reduces the mean duration of severe OM. Our findings do also show that PBM therapy decreases mean pain scores.

PBM is effective in both reducing the grade and duration of OM during the course of chemoradiotherapy in head and neck cancer patients.

Eventually, we can further conclude that the application of PBM accordingly improves QoL of this category of patients since symptoms recover over time of their treatment.

For future research, we advocate to perhaps compare the efficacy of pharmacological agents and PBM. Also, regarding the prophylactic use of PBM, it should be a target to compare PBM therapy at different starting points before the initiation of chemoradiotherapy to avoid unnecessary sessions of PBM
